# Studies on Modulation of Gut Microbiota by Wine Polyphenols: From Isolated Cultures to Omic Approaches

**DOI:** 10.3390/antiox4010001

**Published:** 2015-01-05

**Authors:** Montserrat Dueñas, Carolina Cueva, Irene Muñoz-González, Ana Jiménez-Girón, Fernando Sánchez-Patán, Celestino Santos-Buelga, M. Victoria Moreno-Arribas, Begoña Bartolomé

**Affiliations:** 1 Grupo de Investigación en Polifenoles, Unidad de Nutrición y Bromatología, Facultad de Farmacia, Universidad de Salamanca, Campus Miguel de Unamuno, Salamanca 37007, Spain; E-Mails: mduenas@usal.es (M.D.); csb@usal.es (C.S.-B.); 2 Grupo de Biotecnología Enológica Aplicada, Instituto de Investigación en Ciencias de la Alimentación (CIAL), CSIC-UAM, C/Nicolás Cabrera 9, Campus de Cantoblanco, Madrid 28049, Spain; E-Mails: carolina.cueva@csic.es (C.C.); irene.munoz@csic.es (I.M.-G.); a.jimenez.giron@csic.es (A.J.-G.); fspatan@ifi.csic.es (F.S.-P); mvmoreno@ifi.csic.es (M.V.M.-A.)

**Keywords:** wine, polyphenols, gut microbiota, probiotics, modulation, batch culture fermentation, gastrointestinal simulators, animal models, human studies

## Abstract

Moderate consumption of wine seems to produce positive health effects derived from the occurrence of bioactive polyphenols. The gut microbiota is involved in the metabolism of phenolic compounds, and these compounds and/or their metabolites may modulate gut microbiota through the stimulation of the growth of beneficial bacteria and the inhibition of pathogenic bacteria. The characterization of bacterial metabolites derived from polyphenols is essential in order to understand their effects, including microbial modulation, and therefore to associate dietary intake with particular health effects. This review aims to summarize the current information about the two-way “wine polyphenols–gut microbiota” interaction, from a perspective based on the experimental and analytical designs used. The availability of advanced methods for monitoring bacterial communities, along with the combination of *in vitro* and *in vivo* models, could help to assess the metabolism of polyphenols in the human body and to monitor total bacterial communities, and, therefore, to elucidate the implications of diet on the modulation of microbiota for delivering health benefits.

## 1. Introduction

Wine is considered to be a high bioactive polyphenol content source. Many studies have revealed the key role played by phenolic compounds from grapes and wine on human health; cardiovascular diseases being the pathologies that have received much attention [1,2].

Several studies confirm the importance of the intestinal microbiota to the health of the host, including mental health [3]. Gut bacteria not only help to maximize the absorption of nutrients and energy, but also are essential in the body’s defense mechanisms [4]. Although polyphenol metabolism starts in the mouth and proceeds along the gastrointestinal tract, most of the dietary polyphenols reach the colon, where they are subjected to the action of the gut microbiota, thus releasing aglycones that might, to a certain extent, be absorbed and degraded to simpler phenolic derivatives and other metabolites which could present higher activity at a physiological level than the corresponding food precursors [5]. These metabolites could also be absorbed, increasing polyphenol bioavailability [6]. Non-absorbed polyphenols and/or the resultant phenolic metabolites could affect the growth of gut microbiota, thereby modifying their diversity and metabolic activity. In fact, modulation of gut microbiota by polyphenols has been a topic that has gained increasing attention from the scientific community in recent years, as can be seen from several reviews [7,8,9,10,11,12]. Among the major wine phenolic compounds that may reach the gut, special attention has been given to polymeric flavan-3-ols or proanthocyanidins (also known as condensed tannins) since there is evidence that these polyphenols promote the growth of beneficial bacteria and the inhibition of pathogenic bacteria while they are extensively metabolized by gut microbiota to produce a great range of active metabolites. As relevant reference, an intervention study of cocoa flavan-3-ols in healthy volunteers has shown that they enhance the growth of *Lactobacillus* spp. and *Bifidobacterium* spp. and limit the growth of the *Clostridium histolyticum* group [13]. On the other hand, proanthocyanidins have been found to be largely metabolized into phenylvalerolactones and phenolic acids after cocoa intake in humans and rats [14].

The aim of this review was to summarize the information available on the action of gut microbiota on wine polyphenols (metabolism), as well as the effect of phenolic compounds on the growth of gut bacteria (modulation). This two-way polyphenols–gut microbiota interaction will be assessed from a perspective based on the experimental designs used, from isolated cultures to omic approaches in the case of microbiota analysis, and advanced analytical techniques in the case of metabolite analysis.

## 2. Phenolic Compounds in Wine

**Figure 1:**
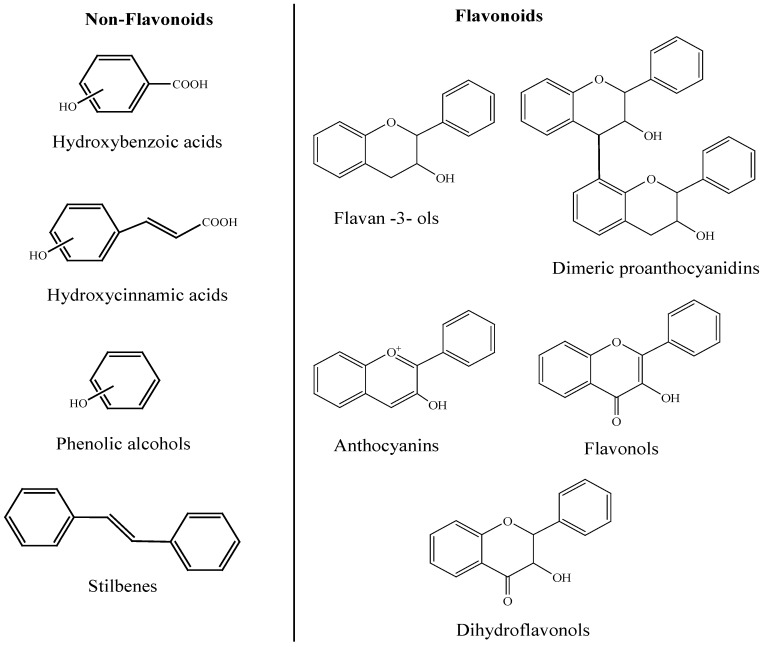
Common phenolic compounds in wine.

The term “phenolic” describes those compounds that possess a benzenic ring substituted by one or several hydroxyl groups (−OH). Polyphenols are secondary metabolites of plants and play an important role in the plant’s defense mechanism against external agents, such as animals or microbial infections, they facilitate pollination and seed dispersion through signals that attract insects and animals, and participate in protection mechanisms against ultraviolet radiation and/or oxidant agents. Phenolic compounds presented in grapes, located in the solid parts of the fruit (skins and seeds), have a wide diversity of chemical structures, including flavonoid compounds (flavan-3-ols (monomers and oligomeric and polymeric proanthocyanidins), anthocyanins, flavonols, and dihydroflavonols) and non-flavonoid compounds (hydroxybenzoic and hydroxycinnamic acids, phenolic alcohols, and stilbenes) (Figure 1). Although the concentration of phenolic compounds in wine is conditioned by several factors related to the grape (variety, soil, geography, climate, *etc.*) and by enological practices, the total polyphenol content is around 50–400 mg/L for white wines, and 900–1400 mg/L for young red wines. Therefore, moderate consumption of wine (250 mL/day) corresponds to an intake of 60 mg of polyphenols for white wines and 210 mg for young red wines [15]. With respect to their distribution by compound groups, acids and hydroxybenzoic derivatives represent approximately 6% of the total; acids and hydroxycinnamic derivatives, 1.1%; stilbenes, 0.5%; alcohols, 3.8%; flavanols, 15%; flavonols, 3.6%; and anthocyanins, 70% in young red wines [15]. Other anthocyanin derivatives such as pyranoanthocyanins present much lower proportions.

Over the last few decades, the role of polyphenols has been the focus of considerable research in the field of nutrition. Several epidemiological studies have shown that the intake of these compounds is inversely associated with the risk of various chronic diseases, such as coronary heart disease, specific cancers, and neurodegenerative disorders [16,17]. Indeed, potential beneficial effects have been demonstrated for different phenolic compounds (especially flavonoids) through *in vitro* assays. In particular, these compounds act as powerful inhibitors of low-density lipoprotein (LDL) oxidation, one of the main mechanisms responsible for the development of atherosclerosis. However, it is currently believed that the physiological activities and mechanisms of these compounds are more diverse and complex. Thus, phenolic compounds are able to inhibit the growth of human cancer cell lines, cholesterol-related processes, and the activity of enzymes, such as telomerase, lipoxygenase and cyclooxygenase involved in inflammatory processes [18,19]. They also interact in different signal transduction pathways, and can affect the cell-cycle regulation, platelet function, and prevent endothelium dysfunction [20,21].

However, the health effects of these compounds depend on their bioavailability, and therefore it is important to understand how they are absorbed, metabolized and eliminated from the body, in order to ascertain their *in vivo* actions.

## 3. General Metabolism of Polyphenols in the Human Body

Polyphenols are considered as xenobiotics by the human organism and therefore are extensively metabolized and finally eliminated, mainly in the bile but also in the urine. The first step in their metabolism is likely to be deglycosylation before absorption in the small intestine. Hydrolysis of some flavonoid glycosides might have already occurred in the oral cavity, as both saliva and oral microbiota show β-glucosidase activity, giving rise to the corresponding aglycones. The hydrolytic activity begins in the mouth, and continues through the digestive tract into the stomach, where the size of food particles is reduced, which prompts the release of phenolic compounds. It has been estimated that 5%–10% of ingested polyphenols are absorbed in the small intestine, while 90%–95% reach the colon where they are intensively degraded by microbiota into a diversity of bioactive phenolic metabolites, lactones and phenolic acids that are then further absorbed [22].

In particular, the glycosylated polyphenols, such as anthocyanins, flavonol glycosides and glycosides of resveratrol, can be hydrolyzed by intestinal β-glucosidases. In contrast, monomeric flavanols, and dimer procyanidins can be absorbed directly into the small intestine. Once absorbed, the resulting aglycones would enter enterocyte by passive diffusion. Thus, the resulting aglycone is rapidly biotransformed by phase II enzymes into conjugated metabolites (*i.e*., glucuronides, *O*-methylethers and/or sulfates) within the enterocyte and again in the liver [23]. Other wine polyphenols, mainly oligomeric flavan-3-ols with a degree of polymerization (mDP) >3 and polymeric flavanols (proantocyanidins and condensed tannins), esters of hydroxycinnamic acids, and flavonols conjugated with rhamnose, such as rutin, are not absorbed in their native forms. These compounds reach the colon, where compounds are subjected to the action of the colonic microflora and transformed into various phenolic acids and other metabolites [24]. The methylated, glucuronide and sulfate conjugates (phase II metabolites) can reach the colon via enterohepatic circulation and are also susceptible to degradation by the intestinal microbiota. Finally, the phenolic metabolites are excreted via urine and feces.

## 4. Gut Microbiota

The orogastrointestinal tract of humans has an abundant microbiota dominated by anaerobic bacteria. The number of bacteria in the oral cavity is about 10^11^ bacteria/g in dental plaque and 10^8^–10^9^ bacteria/mL in saliva, whereas in feces the corresponding numbers are 10^11^–10^12^ bacteria/g [25]. More precisely, the gut microbial ecosystem includes native species that permanently colonize the gastrointestinal tract, and a variable number of live microorganisms that temporarily pass through the digestive tract [4]. Native bacteria are mainly acquired at birth and during the first year of life, whereas transient bacteria are continuously being ingested from food, drinks and the environment.

**Figure 2:**
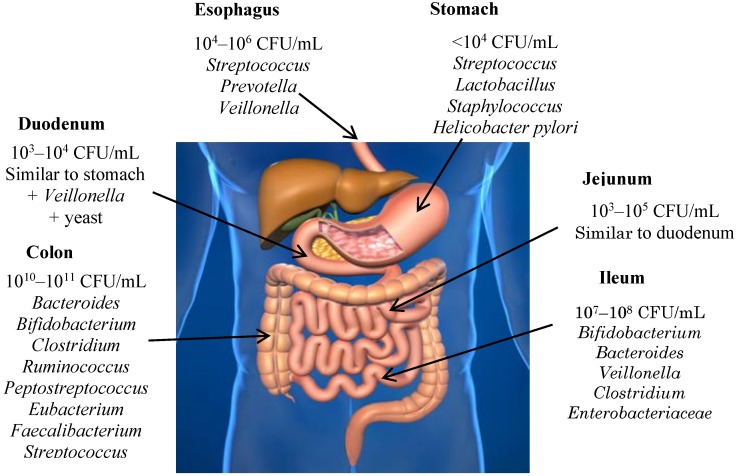
Distribution and composition of bacterial species in the gastrointestinal tract. CFU: colony forming units.

Among the human gastrointestinal microbiota, the majority of the species belong to the phyla Firmicutes, Bacteroides, Actinobacteria and Proteobacteria [26] (Figure 2). The phylogenetic composition of the gut microbiota is considered specific and stable over time for each individual. The species vary greatly between individuals. In fact, interindividual variation in gut microbiota may, in part, reflect differences in dietary intake, although the response of the gut microbiota to dietary change can also differ among individuals. The composition of the individual’s microbiota can fluctuate under some circumstances, for instance acute diarrheal illnesses, antibiotic treatment, or to a lesser extent when being induced by dietary interventions, but the individual flora composition patterns usually remain constant [27]. Among the microbiota associated with the esophagus, are included microorganisms belonging to the genera *Streptococcus*, *Prevotella* and *Veillonella*, which also appear in the oral cavity. The density of colonization is increased about eight times from proximal regions of the small intestine (10^3^ bacterias/g) until the colon. In the stomach and duodenum, the number of microorganisms is reduced due to acid, bile and pancreatic secretions; as advances in the small intestine, the acidity decreases due to the dilution of the acid, which facilitates bacterial colonization, reaching 10^11^ CFU (colony forming units)/mL in the colon. The most frequently found bacteria in this area are members of the genus *Bacteroides*, *Bifidobacterium*, *Eubacterium*, *Clostridium*, *Lactobacillus*, and Gram-positive cocci [28], while *Enterococci* and representatives of the *Enterobacteriaceae* family are found to a lesser extent [29].

Currently, there is evidence that confirms the importance of the gut microbiota in host health is associated to bacterial groups that colonize the intestine. The large intestine contains a complex and dynamic microbial ecosystem composed of commensal bacteria, potentially harmful opportunistic bacteria and others that can have both effects [30]. In particular, having a well-balanced gut microbiota composition is essential for human health. Undesirable bacteria include species of the genus *Clostridium*, *Staphylococcus* and *Veillonella*. These species can produce potentially harmful products, such as toxins and carcinogens, which are associated with intestinal disorders such as chronic inflammatory bowel diseases and other immune-related disorders. However, the use of antibiotics can disrupt the ecological balance and allow the overgrowth of species with potential pathogenicity, such as *Clostridium difficile*, associated with pseudomembranous colitis [31]. With regards to beneficial bacteria, among which are mainly included species of the genus *Lactobacillus* and *Bifidobacterium*, these play a key role in nutritional and disease-prevention functions, so they are used as probiotics. The main functions of these bacteria are: decreasing gas production, the production of short-chain fatty acids (SCFA), immunostimulating and antitumor activity [32,33].

Although the investigations concerning the influence of polyphenols on the gut microbiota and their mechanisms of action in humans are scarce, nevertheless drastic changes in fecal- and mucosa-associated microbiota could be related to determined diseases, such as colon cancer, short bowel syndrome and obesity [34]. It is important, therefore, to investigate gut microbiota composition and the influence of dietary intake on the human microbiome, to elucidate the implications of diet on the modulation of microbiota for delivering health benefits.

The availability and feasibility of advanced methods for monitoring total bacterial communities is a prerequisite to be considered when designing a study to assess gut microbiota. Over recent decades, the introduction of culture-independent techniques like terminal restriction fragment length polymorphism (T-RFLP), fluorescence *in situ* hybridation (FISH), denaturing gradient gel electrophoresis (DGGE), and quantitative polymerase chain reaction (qPCR) have improved the analysis of gut microbiota. At present, next-generation sequencing (NGS) techniques have promoted the emergence of new, high-throughput technologies, such as genomics, metagenomics, transcriptomics, metatranscriptomics, *etc.* The development of these techniques has provided the opportunity to explore the taxonomic, protein-coding gene or expression diversity by applying more comprehensive and less biased measurements to all systems involved (*i.e.*, diet, microbiota and host) [35]. However, the enormous amount of data generated becomes cumbersome to analyze, requiring much dedicated time as well as expertise to manage data in such quantity [36]. The link between high-throughput qPCR and next generation sequencing technologies provides manageable data with valuable quantitative and taxonomic information. NGS platforms involve many different technologies [37] all of which generate large, genome-scale datasets. Examples of current commercial platforms are the 454 (Roche), Solexa (Illumina), SOLiD and Ion Torrent (Life Technologies), and PacBio (Pacific Biosciences) systems.

## 5. Interaction between Wine Polyphenols and Gut Microbiota

Taking the above-mentioned points into account, it is evident that the gut microbiome is implicated in the metabolism of wine polyphenols, and it also suggests that phenolic compounds and their metabolites may modulate the gut microbiota to a certain extent. The so-called two-way interaction between wine polyphenols and gut microbiota [7] can be subjected to various factors related to the phenolic compounds concentration and structure as well as the characteristics of bacterial strains, microbial environment, food matrix, *etc.* Prior to the specific sections about wine polyphenol metabolism and bacterial modulation by polyphenols, a preliminary section about experimental and analytical approaches used in these studies is included.

### 5.1. Experimental Designs and Analytical Techniques

#### 5.1.1. *In Vitro* Gastrointestinal Tract Simulators

Studies regarding polyphenol metabolism and microbial modulation have been carried out using different experimental designs, from simple approaches involving fermentation experiments in batch cultures and in continuous simulators, to intervention trials in animals and humans. Although *in vivo* trials are most relevant physiologically, *in vitro* tools have been designed to simulate intestinal conditions and the transformations undergone in foods during transit through the gastrointestinal tract.

Simply, static gut models, also known as batch-type cultures, are generally closed systems using sealed bottles or reactors containing suspensions of fecal material, which are maintained under anaerobic conditions. This model approach is primarily used when assessing the stability of polyphenols in the presence of human-derived gut microbiota and evaluating which environmental conditions favor or limit polyphenol bioconversion.

In contrast to short-duration experiments with static gut models, longer-term experiments are required when the adaptation of the gut microbial community to dietary polyphenols needs to be assessed. To that end, simulators of the gastrointestinal tract comprise stomach and small intestinal sections for the pre-digestion of food as well as vessels stimulating the ascending, transverse and descending sections of the human colon, allowing the assessment of changes in colonic areas that are very challenging to access in a human intervention, as well as the physiological variables (*i.e.*, retention time, pH variations, gastrointestinal fluids, microbiota).

In the past few years, dynamic *in vitro* gut models, such as SHIME (simulator of the human intestinal microbial ecosystem) (University of Ghent, Belgium) [38], the TIM-1 and TIM-2 (developed by TNO, Netherlands Organization for Applied Scientific Research, The Netherlands) [39] and the recent SIMGI (dynamic gastrointestinal tract simulator) (CIAL, CSIC-UAM, Spain), have been developed to simulate physiological conditions that can have an influence on the gut microbiota and their metabolic activity. All these systems contain different compartments representing the stomach, small intestine and colon.

#### 5.1.2. Analytical Approaches to the Analysis of Phenolic Microbial-Derived Metabolites

Rapid, sensitive, and reliable analytical methods are needed for profiling microbe-derived phenolic metabolites in biological fluids in order to determine their contribution to the overall bioavailability of polyphenols and to allow identification of biomarkers of polyphenol exposure that could be further used to correlate dietary intake with particular health effects, including microbial modulation.

Gas chromatography (GC) coupled to mass spectrometry techniques (MS) and liquid chromatography (LC) coupled to a diode array detector (DAD), electrochemical (ECD) and, in particular, tandem mass spectrometry (ESI-MS/MS) are the most widely used analytical techniques for the analysis of microbial phenolic metabolites in biological fluids, which can be found in micromolar concentration. GC methodologies provide higher resolution and sensitivity than LC methodologies but a laborious sample preparation stage is required, since they usually involve the isolation of metabolites by liquid-liquid extraction (LLE) or other extraction procedures followed by further derivatization [40]. In contrast, for LC analysis the sample preparation step is more simple and faster and provides a very sensitive method for the quantification of selected phenolic metabolites when coupled to MS/MS [41]. The efficiency of separation and the sensitivity of LC–MS/MS methodologies can be largely improved by the use of ultra performance liquid chromatography (UPLC), which operates with smaller particle size (<2 μm) sorbent materials and at very high pressures (up to 15,000 psi). This technique coupled to MS detection is considered a good application in the analysis of phenolic compounds in biological fluids and food matrixes [41].

Metabolomics approaches have been used in clinical, pharmaceutical, and toxicological applications and have also recently emerged as fields of increasing importance in food and nutrition sciences [42]. The metabolome can be affected by different external and internal factors. Among them, diet is a very important external factor affecting the urinary metabolome because it produces notable changes in its composition [43]. Metabolomics studies of the polyphenols may lead to the discovery of new phytochemical metabolites and new biomarkers of intake that could allow the intake of dietary phytochemicals to be monitored, and eventually relate them to the expected biological effects [44].

### 5.2. Biotransformation of Wine Polyphenols by Gut Microbiota

**Figure 3:**
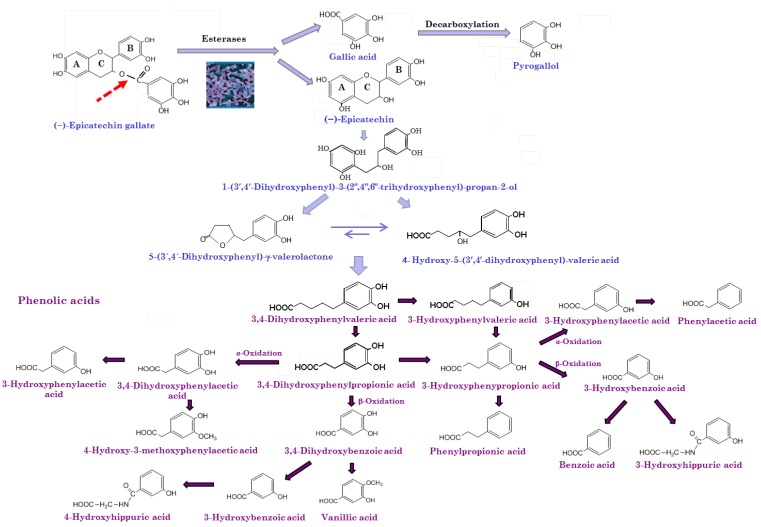
Catabolism of monomeric flavan-3-ols by gut microbiota.

The microbial biotransformation of wine polyphenols is widely influenced by their chemical structure. Oligomers and polymers of flavan-3-ols are the major phenolic compounds present in the wine that reaches the colon. The colonic metabolism of dimeric procyanidins involves C-ring opening, followed by lactonization, decarboxylation, dehydroxylation, and oxidation reactions, among others [45] (Figure 3). In the case of galloylated monomeric flavan-3-ols, the microbial metabolism usually starts with the rapid cleavage of the gallic acid ester moiety by microbial esterases, giving rise to gallic acid which is further decarboxylated into pyrogallol. The C-ring is subsequently opened, giving rise to 1-(3′,4′-dihydroxyphenyl)-3-(2″,4″,6″-trihydroxyphenyl)-propan-2-ol, which is later converted into 5-(3′,4′-dihydroxyphenyl)-γ-valerolactone in the case of (epi)catechin or 5-(3′,4′,5′-trihydroxyphenyl)-γ-valerolactone in the case of (epi)gallocatechin. The valerolactone ring later breaks, giving rise to 5-(3′,4′-dihydroxyphenyl)valeric acid and/or 4-hydroxy-5-(3′,4′-dihydroxyphenyl) valeric acid. Subsequent biotransformations of these valeric acids give rise to hydroxyphenylpropionic and hydroxybenzoic acids by successive loss of carbon atoms from the side chain through β-oxidation (Figure 3).

With regard to microbial catabolism of flavonols, other flavonoid compounds present in the wine are directly transformed into 3,4- or 3,5-dihydroxylated phenylacetic acids [46]. In the case of anthocyanins, they are converted into 3,4-dihydroxy-, 4-hydroxy-, 3,4-dimethoxy- or 3-methoxyl-4-hydroxyl benzoic acids according to the substitution pattern of the B-ring of the precursor anthocyanin molecule [46]. However, in spite of the fact that anthocyanins are abundant in wine, their circulating levels in plasma are very low, which has been attributed to anthocyanin instability under neutral pH [47], their extensive metabolism *in vivo* [48], and their probable catabolism by intestinal microbiota [5]. Thus, it has been described that less than 1% of the ingested anthocyanins are typically absorbed and excreted in the urine [49], which might be found in low concentrations in serum, feces, urine (in the nanomolar concentrations) [50].

In the case of non-flavonoid compounds present in wine, hydroxycinnamic esters (*i.e.*, caffeic acid derivatives) are mainly transformed into 3-hydroxyphenylpropionic acid, benzoic acid and 4-ethylcatechol. Once absorbed, the microbial metabolites are mainly metabolized in the liver by phase II enzymes as conjugated metabolites (glucuronides and sulfates). Other reactions that can take place are glycination, dehydrogenation, hydroxylation and methylation. In fact, different studies have proved the presence of phase II and microbial-derived phenolic metabolites in plasma and urine after ingestion of wine and wine polyphenols (see for review [51,52]). Some previous studies have confirmed that a moderate intake of red wine effectively led to a significant increase of phenolic metabolites in human feces [53,54].

Despite the advances recently made in the knowledge of the identification of phenolic metabolites, the specific bacterial species able to metabolize most wine polyphenols in the gastrointestinal tract and the anaerobic degradation pathways have still not been identified. So far, only a few bacterial species, some of them belonging to the class *Clostridiales*, such as *Eubacterium* sp. and *Flavonifractor* sp., and *Eggerthella* spp., have been reported to be able to initiate the metabolism of flavanol-3-ols [55,56,57].

### 5.3. Modulation of Gut Microbiota by Wine

Table 1 and Table 2 report different studies of modulation of gut microbiota by wine and grape polyphenols using different experimental approaches: batch culture fermentations, human gastrointestinal simulators, animal model studies and human interventions. For all of them, details about experimental conditions and/or study design, microbial techniques used and main effects on bacteria groups (growth enhancement, growth inhibition or no effect) are included.

**Table 1:** Studies regarding modulation of gut microbiota by wine polyphenols using batch culture fermentations and gastrointestinal tract simulator.

Studies Using Batch Culture Fermentation								
Reference	Fecal Concentration	Phenolic Compound/Food	Dose	Time of Incubation	Microbial Technique	Growth Enhancement	Growth Inhibition	No Effect
[58]	10%, w/v	(+)-Catechin	150 mg/L, 1000 mg/L	<48 h	FISH	*Lactobacillus*–*Enterococcus* spp.; *Bifidobacterium* spp.; *C. coccoides*–*E. Rectale* group *E. coli*	*C. histolyticum* group	
[59]	10%, w/v	Malvidin-3-*O*-glucoside Anthocyanidins mixture	20 mg/L and 200 mg/L 4850 mg/L and 48,500 mg/L	<24 h	FISH	*Lactobacillus*–*Enterococcus* spp.; *Bifidobacterium* spp.; *C. coccoides*–*E. rectale* group		
[60]	10%, w/v	Grape seed extract fractions	300–450 mg/L	<48 h	FISH	*Lactobacillus*–*Enterococcus* spp.	*C. histolyticum* group	
[61]	1% w/v	Red wine extract	600 mg/L	48 h	FISH		*C. histolyticum* group	*Lactobacillus*–*Enterococcus spp.*
[62]		Red wine extract	500 mg/L	48 h	qPCR	*Lactobacillus* spp.; *Bifidobacterium* spp.; *Bacteroides* spp.; *Ruminococcus* spp.		
[63]	20% w/v	Red wine/grape extract	500–1000 mg/L	72 h	HITChip			
**Studies Using a Gastrointestinal Simulator**								
**Reference**	**Simulator**	**Phenolic Compound/Food**	**Dose**	**Time**	**Microbial Technique**	**Population Increase**	**Population Decrease**	**No Effect**
[64]	Twin-SHIME	Red wine-grape extract	3 × daily dosing (1000 mg polyphenols as total daily dose)	2 weeks	Plate count qPCR PCR-DGGE; Pyrosequencing	*Klebsiella* spp.; *Alistipes* spp.; *Cloacibacillus* spp.; *Victivallis* spp.; *Akkermansia* spp.	Bifidobacteria; *Blautia coccoides* group; *Anaeroglobus* spp.; *Subdoligranulum* spp. *Bacteroides*	

**Table 2:** Studies regarding modulation of gut microbiota by wine polyphenols in studies with animals and humans.

Animal Model Studies									
Reference	Simulator	Phenolic Compound/Food		Dose	Time	Microbial Technique	Population Increase	Population Decrease	No Effect
[65]	Rats	Red wine polyphenols powder		50 mg/kg	16 weeks	Plate count	Lactobacilli; Bifidobacteria	Propionibacteria; *Bacteroides*; Clostridia	
[66]	Broiler chicks	Grape seed extract (GSE)		7.2 g/kg diet (GSE) (free access)	21 days	Plate count T-RFLP	*E. coli; Enterococcus* spp.; *Lactobacillus* spp.		
[67]	Pigs	Grape seed extract		1% (free access)	4 weeks	qPCR		*Streptococcus* spp.; *Clostridium* Cluster XIVa	*Lactobacillus* spp; *Bifidobacterium* spp.
[68]	Pigs	Grape seed extract		1% w/w	6 days	Ilumina MiSeq platform	*Lachnospiraceae*, *Clostridales*, *Lactobacillu*, *Ruminococcacceae*		
**Human Intervention Studies**									
**Reference**	**Volunteer Numbers**	**Phenolic Compound/Food**	**Dose**		**Treatment Duration**	**Microbial Technique**	**Population Increase**	**Population Decrease**	**No Effect**
[69]	9	Proantocyanidin-rich extract from grape seeds	0.5 g/day		6 weeks	Plate count	*Bifidobacterium* spp.	*Enterobacteriaceae*	
[70]	10	Red wine	272 mL/day		20 days	qPCR	*Enterococcus* spp.; *Prevotella* spp.; *Bacteroides Bifidobacterium* spp.; *Bacteroides uniformis Eggerthella lenta Blautia coccoides*–*E. rectale* group	*Clostridium* spp.; *C. histolyticum* group	Actinobacteria

#### 5.3.1. Studies Using Batch Culture Fermentations

One of the first relevant experiments using batch culture fermentation and a standard compound present in grape seeds and wine was carried out by Tzounis *et al.* [58] who found that the flavan-3-ol monomers ((−)-epicatechin and (+)-catechin) promoted the growth of the *Clostridium coccoides*–*Eubacterium rectale* group, which is known to produce large amounts of butyrate, a short-chain fatty acid (SCFA) with anti-inflammatory and antineoplastic properties (Table 1). Compared to (−)-epicatechin, (+)-catechin exposure resulted in a greater modification of the growth of the bacterial groups; (+)-catechin also increased the growth of *Lactobacillus*–*Enterococcus* spp., *Bifidobacterium* spp. and *Escherichia coli* but decreased the growth of *Clostridium histolyticum*. In contrast, the effect of (−)-epicatechin only significantly increased the growth of the *C. coccoides*–*E. rectale* group. Also using standard compounds of anthocyanins (*i.e.*, malvidin-3-glucoside and a mixture of anthocyanins), Hidalgo *et al.* [59] found a significant increase in the growth of *Lactobacillus*–*Enterococcus* spp. and *Bifidobacterium* spp.

Recently, our research group carried out several batch culture fermentations of two flavan-3-ol fractions with different degrees of polymerization from grape seed extract and of wine polyphenols, with fecal microbiota from three healthy volunteers [60,61]. Both flavan-3-ol fractions induced the growth of *Lactobacillus/Enterococcus* spp. and inhibited the *C. histolyticum* group during fermentation, although the effects were only statistically significant with the less polymerized fraction. However, wine polyphenols only showed a slight inhibition in the *C. histolyticum* group, probably due to their lower content in flavan-3-ols and the time of exposure. Additionally, this type of fermentation has also been used to assess the contribution of certain probiotic strains to the colonic metabolism of polyphenols. With this in mind, Barroso *et al.* [62] carried out fermentations of a red wine extract inoculated with human microbiota obtained from the colonic compartments of a dynamic simulator, in the presence and absence of the probiotic strain *Lactobacillus plantarum* IFPL935. Microbial analysis by qPCR indicated that red wine polyphenols induced greater variations among *in vitro* batches harboring different colon-region (ascending colon, descending colon and effluent) microbiota than those found when *L. plantarum* IFPL935 was added. Batches inoculated with microbiota from the ascending colon were shown to harbor the major proportion of saccharolytic bacteria (*Bacteroides*, *Bifidobacterium*, *Prevotella*) whereas *Clostridium* groups were found in major numbers in the batches inoculated with microbiota simulating the distal regions [62].

Gross *et al.* [63] carried out *in vitro* fermentation of a red wine/grape and black tea polyphenols with fecal samples from 10 human volunteers. Microbial composition of fecal samples using HITChip analysis indicated different bacterial populations present in the individual fecal microbial communities. This inter-individual variability was related to the production of different metabolites, suggesting that each individual generates a specific microbial metabolome.

#### 5.3.2. Studies Using Human Gastrointestinal Simulators

As an example of the versatility and potential of human gastrointestinal simulators, Table 1 also reports a study concerning the modulation of gut microbiota by polyphenols using the SHIME [38]. This validated model comprises stomach and small intestinal sections for predigestion of food as well as vessels stimulating the ascending, transverse and descending parts of the human colon, allowing assessment of changes in the different colonic areas that are very challenging to access in a human intervention.

The use of the twin-SHIME to investigate the effects of a red wine extract on the colonic microbiota was the aim of a study recently carried out by Kemperman *et al.* [64]. These authors characterized microbial community changes using a combination of analyses including cultivation, PCR-denaturing gradient gel electrophoresis (DGGE), quantitative PCR and high-throughput pyrosequencing of the 16S ribosomal RNA gene. They observed that the continuous administration of red wine extracts for two weeks could modulate select members of the human gut microbiota, revealing novel targets potentially involved in polyphenol metabolism and/or resistant microbes.

#### 5.3.3. Animal Model Studies

It is widely known that preliminary evidence should be warranted in animal models before human intervention trials. Animal models contribute to a better understanding of the mechanisms and biological effects that could be likely to happen in the human body. Caution is required in extrapolating results to humans because culture-independent comparisons have revealed that most bacterial genera and species found in mice are not seen in humans, although the distal gut microbiota of mice and humans harbor the same bacterial phyla. Nevertheless, the use of germ-free mice inoculated with human fecal microbiota is of great relevance. Such humanized mice provide a model system for controlling host genotype, gut microbiota composition, diet, and housing conditions. To date, no studies have followed the composition of the intestinal microbiota in humanized rodents fed a diet rich in wine polyphenols [71].

In this section, studies performed on animals in order to assess the effects of wine polyphenols on the modulation of intestinal microbiota are summarized (Table 2).

Dolara *et al.* [65] showed that treatment with wine polyphenols in carcinogen-treated F344 rats was associated with a strong variation in the colonic microbiota, compared to the control-fed rats. Although the total bacterial counts and anaerobe/aerobe ratio of microorganisms in the feces from polyphenol-treated rats were similar to those from control rats, Propionibacteria, *Bacteroides* and *Clostridia* decreased while Lactobacilli and Bifidobacteria increased. Based on additional experiments, these authors concluded that reduction of oxidative damage, modulation of colonic flora and variation in gene expression may all be connected in the action of wine polyphenols on the intestinal function and carcinogenesis.

Another animal experiment was conducted to study the effect of the inclusion of grape seed extracts in the diet of broiler chicks [66] on intestinal microbiota. It was observed that, for the cecum, birds fed grape extracts had higher populations of *E. coli*, and *Lactobacillus* and *Enterococcus* species than birds in control groups. These authors concluded that polyphenol-rich grape products modified the gut morphology and intestinal microbiota and increased the degree of biodiversity in intestinal bacteria of broiler chicks.

Animal studies performed in pigs [67,68] demonstrated that grape seed extract administration caused an ecological shift in the microbiome. Thus, the administration of grape seed extracts produced lower counts of *Streptococcus* spp and *Clostridium* cluster XIVa [67], and increased *Lachnospiraceae*, *Clostridales*, *Lactobacillus* and *Ruminococcus* during the intervention period [68].

#### 5.3.4. Human Intervention Studies

Investigations carried out with humans potentially provide the best models for studying the interactions of food components (e.g., polyphenols) with microbiota, although *in vivo* intervention trials hold inevitable practical and ethical limitations [8]. The use of crossover designs, where volunteers serve as their own control, permit multilevel analysis schemes that increase power, but as with other human intervention studies, they require a relevant number of volunteers to allow for statistically significant multivariate models [72]. Up to now, only a few studies have examined the *in vivo* impact of dietary polyphenols on the human gut microbiota, and most of them were focused on single polyphenol molecules and selected bacterial populations (Table 2).

In a study with a reduced number of subjects (*n* = 9), Yamakoski *et al.* [69] reported that administration of a proanthocyanidin-rich extract from grape seed significantly increased the fecal number of *Bifidobacterium* spp., whereas a reduction in the level of putrefactive bacteria, such as *Enterobacteriaceae* was observed.

Queipo-Ortuño *et al.* [70] performed a randomized, crossover and controlled trial (*n* = 10), which studied the effect of the intake of red wine, de-alcoholized red wine and gin over three consecutive periods. After the red wine period, the bacterial concentrations of *Proteobacteria*, *Fusobacteria*, *Firmicutes* and *Bacterioidetes* markedly increased compared with the washout period; significant increases in the number of *Bifidobacterium* spp. and *Prevotella* spp. were also observed. In contrast, *Clostridium* spp. and *C. histolyticum* group concentrations decreased after the red wine period.

## 6. Conclusions

Assessment of microbial metabolism of wine polyphenols is necessary to clarify the potential physiological effects on human health associated with moderate consumption of wine. There is scientific evidence that polyphenols may contribute to the maintenance of gut health by the modulation of the gut microbial balance through the stimulation of the growth of beneficial bacteria and the inhibition of pathogen bacteria, exerting prebiotic-like effects. Although genetic and environmental factors are the main determinants of gut microbiota composition, it is well established that diet influences microbial fermentation and total bacteria in the intestine. In fact, inter-individual variation in gut microbiota may, in part, reflect differences in dietary intake, although the response of the gut microbiota to dietary change can also differ among individuals. Therefore, the availability of advanced methods for monitoring bacterial communities could provide a better understanding of the underlying mechanisms in the polyphenols–microbiota–host triangle, and elucidate the implications of polyphenols on host health.
